# Aqua[*N*-(2,5-dihydroxybenzyl)imino­diacetato]copper(II)

**DOI:** 10.1107/S1600536809042238

**Published:** 2009-10-17

**Authors:** Xiu-Qing Zhang, Fu-Ping Huang, He-Dong Bian, Qing Yu, Hong Liang

**Affiliations:** a Key Laboratory for the Chemistry and Molecular Engineering of Medicinal Resources (Ministry of Education of China), School of Chemistry and Chemical Engineering of Guangxi Normal University, Guilin 541004, People’s Republic of China

## Abstract

The title complex, [Cu(C_11_H_11_NO_6_)(H_2_O)], contains a Cu^II^ atom in a distorted square-pyramidal geometry. The metal centre is coordinated in the basal sites by one water mol­ecule and two carboxyl­ate O atoms and one N atom of the tetra­dentate ligand [Cu—O range, 1.9376 (11)–1.9541 (12), Cu—N, 1.9929 (12) Å] while the apical site is occupied by a hydro­quinone O donor atom [Cu—O, 2.3746 (12) Å]. Inter­molecular hydrogen bonding inter­actions involving both hydro­quinone hydr­oxy groups and the coordinated water as donors give a three-dimensional framework structure.

## Related literature

For general background to *p*-hydro­quinones and their oxidation products *p*-semiquinones and *p*-quinones, see: Dooley *et al.* (1998 ▶); Wang *et al.* (1996 ▶); Calvo *et al.* (2000 ▶); Iwata *et al.* (1998 ▶); Drouza *et al.* (2002 ▶); Huang *et al.* (2008 ▶); Addison *et al*. (1984 ▶). For the synthesis, see: Fan (1992 ▶). 
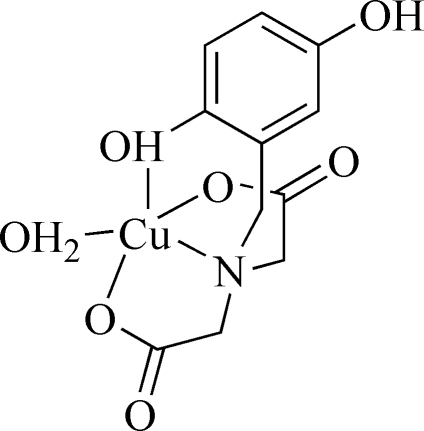


## Experimental

### 

#### Crystal data

[Cu(C_11_H_11_NO_6_)(H_2_O)]
*M* *_r_* = 334.76
Orthorhombic, 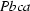

*a* = 13.0461 (16) Å
*b* = 9.7919 (12) Å
*c* = 19.374 (2) Å
*V* = 2474.9 (5) Å^3^
*Z* = 8
Mo *K*α radiation
μ = 1.80 mm^−1^
*T* = 294 K
0.22 × 0.18 × 0.12 mm

#### Data collection

Rigaku Saturn diffractometer
Absorption correction: multi-scan (Jacobson, 1998 ▶) *T*_min_ = 0.693, *T*_max_ = 0.813
17710 measured reflections
2929 independent reflections
2662 reflections with *I* > 2σ(*I*)
*R*_int_ = 0.026

#### Refinement

*R*[*F* ^2^ > 2σ(*F* ^2^)] = 0.025
*wR*(*F* ^2^) = 0.070
*S* = 1.04
2929 reflections
198 parameters
H atoms treated by a mixture of independent and constrained refinement
Δρ_max_ = 0.31 e Å^−3^
Δρ_min_ = −0.56 e Å^−3^

### 

Data collection: *CrystalClear* (Rigaku/MSC, 2005 ▶); cell refinement: *CrystalClear*; data reduction: *CrystalClear*; program(s) used to solve structure: *SHELXS97* (Sheldrick, 2008 ▶); program(s) used to refine structure: *SHELXL97* (Sheldrick, 2008 ▶); molecular graphics: *SHELXL97*; software used to prepare material for publication: *CrystalStructure* (Rigaku/MSC, 2005 ▶).

## Supplementary Material

Crystal structure: contains datablocks I, global. DOI: 10.1107/S1600536809042238/zs2013sup1.cif

Structure factors: contains datablocks I. DOI: 10.1107/S1600536809042238/zs2013Isup2.hkl

Additional supplementary materials:  crystallographic information; 3D view; checkCIF report

## Figures and Tables

**Table 1:** Hydrogen-bond geometry (Å, °)

*D*—H⋯*A*	*D*—H	H⋯*A*	*D*⋯*A*	*D*—H⋯*A*
O5—H5⋯O2^i^	0.76 (3)	1.93 (3)	2.6859 (16)	172 (3)
O6—H4⋯O3^ii^	0.86 (3)	2.00 (3)	2.8442 (18)	168 (2)
O7—H7*A*⋯O2^iii^	0.80 (2)	1.97 (2)	2.7261 (16)	157 (2)
O7—H7*B*⋯O4^iv^	0.87 (3)	1.83 (3)	2.6794 (18)	163 (2)

Symmetry codes: (i) 
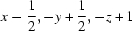
; (ii) 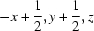; (iii) 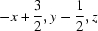; (iv) 
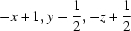
.

**Fig. 1.**
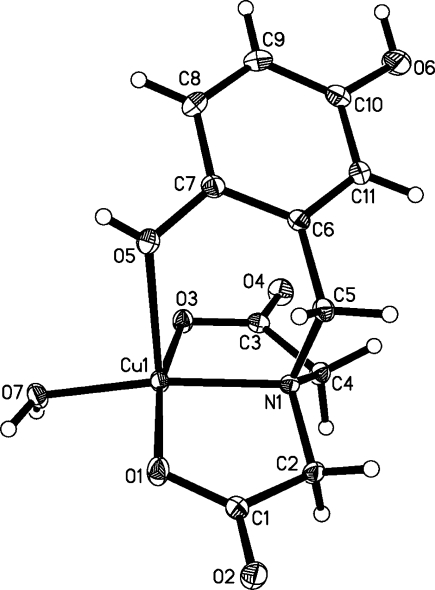
A view of the molecular structure of (I) with the atom-numbering scheme and 30% displacement ellipsoids.

**Fig. 2.**
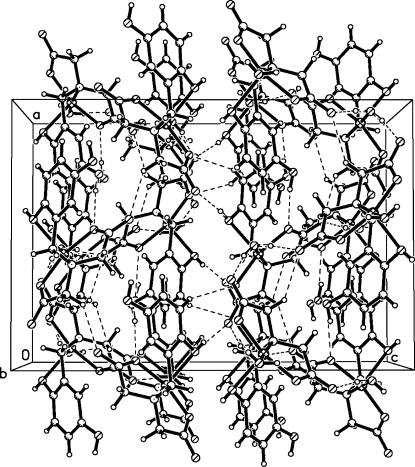
The packing for (I), showing hydrogen bonds as dashed lines.

## supplementary crystallographic information

### Comment

*p*-Hydroquinones, along with their oxidation products *p*-semiquinones and *p*-quinones, are very important in the oxidative maintenance of biological amine levels (Dooley *et al.*, 1998), tissue formation (Wang *et al.*, 1996), photosynthesis (Calvo *et al.*, 2000) and aerobic respiration (Iwata *et al.*, 1998). These compounds are involved in interesting organic electron- and hydrogen-transfer systems, e.g. electron-transfer reactions between transition metal centers and *p*-quinone cofactors are vital for all life (Drouza *et al.*, 2002), occurring in key biological processes. As part of a series of the studies (Huang *et al.*, 2008), we report here the synthesis and structure of the title compound, a new Cu^II^ complex with the related ligand 2-[*N*,*N*-bis(carboxylatomethyl)aminomethyl]hydroquinone. The molecular structure of the title compound [Cu(C_11_H_11_NO_6_)(H_2_O)] (I) is shown in Fig. 1. The Cu^II^ atom has a distorted square-pyramidal geometry with a τ parameter of 0.09 (Addison *et al.*, 1984). The basal sites are occupied by one water molecule, as well as two carboxylate O atoms and one N atom of the ligand. In the apical position, the O atom of the hydroxybenzene coordinates to the Cu^II^ atom. All bond distances and bond angles have normal values. The crystal packing of (I) (Fig. 2) involves intermolecular O—H···O hydrogen bonds (Table 1). The non-coordinated carboxylate O2 atom accepts intermolecular hydrogen bonds from the coordinated hydroxy O (O5) of the hydroquinone ligand and from the coordinated water (O7). The non-coordinated carboxylate O4 atom is also an acceptor for a water H donor in an intermolecular hydrogen bond. The coordinated atom O3 accepts a hydrogen bond from the non-coordinated hydroquinone O (O6). These interactions result in a three-dimensional hydrogen-bonded framework structure.

### Experimental

The ligand 2-[*N*,*N*-bis(carboxylatomethyl)aminomethyl]hydroquinone was prepared according to a literature procedure (Fan *et al.*, 1992). The title complex was synthesized by the addition of CuCl_2_.2H_2_O (0.0850 g, 0.5 mmol) to 20 ml of a methanol solution containing the ligand (0.1275 g, 0.5 mmol). The resulting solution was stirred for 3 h at 60°C, and then cooled and filtered. Blue single crystal blocks were isolated from the solution at room temperature over six days.

### Refinement

H atoms on C atoms were positoned geometrically with C—H_aromatic_ = 0.93 Å and C—H_aliphatic_ = 0.97 Å and treated as riding with *U*_iso_(H) = 1.2*U*_eq_(C).

### Crystal data

**Table:**

[Cu(C_11_H_11_NO_6_)(H_2_O)]	*F*(000) = 1368
*M**_r_* = 334.76	*D*_x_ = 1.797 Mg m^−^^3^
Orthorhombic, *P**b**c**a*	Mo *K*α radiation, λ = 0.71070 Å
Hall symbol: -P 2ac 2ab	Cell parameters from 6907 reflections
*a* = 13.0461 (16) Å	θ = 1.6–27.9°
*b* = 9.7919 (12) Å	µ = 1.80 mm^−^^1^
*c* = 19.374 (2) Å	*T* = 294 K
*V* = 2474.9 (5) Å^3^	Block, blue
*Z* = 8	0.22 × 0.18 × 0.12 mm

### Data collection

**Table:**

Rigaku Saturn diffractometer	2929 independent reflections
Radiation source: rotating anode	2662 reflections with *I* > 2σ(*I*)
confocal	*R*_int_ = 0.026
Detector resolution: 7.31 pixels mm^-1^	θ_max_ = 27.9°, θ_min_ = 2.1°
ω scans	*h* = −17→17
Absorption correction: multi-scan (Jacobson, 1998)	*k* = −12→11
*T*_min_ = 0.693, *T*_max_ = 0.813	*l* = −25→22
17710 measured reflections	

### Refinement

**Table:**

Refinement on *F*^2^	Primary atom site location: structure-invariant direct methods
Least-squares matrix: full	Secondary atom site location: difference Fourier map
*R*[*F*^2^ > 2σ(*F*^2^)] = 0.025	Hydrogen site location: inferred from neighbouring sites
*wR*(*F*^2^) = 0.070	H atoms treated by a mixture of independent and constrained refinement
*S* = 1.04	*w* = 1/[σ^2^(*F*_o_^2^) + (0.0451*P*)^2^ + 0.7209*P*] where *P* = (*F*_o_^2^ + 2*F*_c_^2^)/3
2929 reflections	(Δ/σ)_max_ = 0.001
198 parameters	Δρ_max_ = 0.31 e Å^−^^3^
0 restraints	Δρ_min_ = −0.56 e Å^−^^3^

### Special details

**Table:**

Geometry. All e.s.d.'s (except the e.s.d. in the dihedral angle between two l.s. planes) are estimated using the full covariance matrix. The cell e.s.d.'s are taken into account individually in the estimation of e.s.d.'s in distances, angles and torsion angles; correlations between e.s.d.'s in cell parameters are only used when they are defined by crystal symmetry. An approximate (isotropic) treatment of cell e.s.d.'s is used for estimating e.s.d.'s involving l.s. planes.
Refinement. Refinement of *F*^2^ against ALL reflections. The weighted *R*-factor *wR* and goodness of fit *S* are based on *F*^2^, conventional *R*-factors *R* are based on *F*, with *F* set to zero for negative *F*^2^. The threshold expression of *F*^2^ > σ(*F*^2^) is used only for calculating *R*-factors(gt) *etc*. and is not relevant to the choice of reflections for refinement. *R*-factors based on *F*^2^ are statistically about twice as large as those based on *F*, and *R*- factors based on ALL data will be even larger.

### Fractional atomic coordinates and isotropic or equivalent isotropic displacement parameters (Å^2^)

**Table:**

	*x*	*y*	*z*	*U*_iso_*/*U*_eq_	
Cu1	0.566322 (13)	0.226573 (19)	0.381922 (9)	0.01771 (8)	
O1	0.68631 (9)	0.23223 (12)	0.44079 (6)	0.0273 (3)	
O2	0.83131 (8)	0.34779 (12)	0.45719 (6)	0.0275 (3)	
O3	0.47226 (9)	0.23658 (11)	0.30369 (5)	0.0214 (2)	
O4	0.43813 (9)	0.36157 (13)	0.21087 (6)	0.0300 (3)	
O5	0.43629 (9)	0.31326 (14)	0.45565 (6)	0.0259 (3)	
H5	0.402 (2)	0.269 (3)	0.4779 (13)	0.049 (7)*	
O6	0.24306 (11)	0.69678 (15)	0.29249 (7)	0.0402 (3)	
H4	0.179 (2)	0.700 (3)	0.3008 (12)	0.046 (6)*	
O7	0.54070 (9)	0.03204 (12)	0.39637 (7)	0.0242 (2)	
H7A	0.5782 (16)	−0.006 (2)	0.4227 (12)	0.039 (6)*	
H7B	0.5424 (18)	−0.010 (3)	0.3566 (13)	0.046 (7)*	
N1	0.60886 (9)	0.41382 (12)	0.35356 (6)	0.0164 (2)	
C1	0.74866 (11)	0.32885 (16)	0.42719 (7)	0.0205 (3)	
C2	0.72035 (11)	0.42214 (16)	0.36733 (8)	0.0225 (3)	
H2A	0.7389	0.5155	0.3784	0.027*	
H2B	0.7581	0.3952	0.3264	0.027*	
C3	0.48912 (11)	0.33842 (16)	0.26298 (7)	0.0200 (3)	
C4	0.58171 (11)	0.42657 (17)	0.27948 (7)	0.0209 (3)	
H4A	0.6394	0.3983	0.2513	0.025*	
H4B	0.5665	0.5212	0.2688	0.025*	
C5	0.55454 (11)	0.51871 (16)	0.39631 (8)	0.0212 (3)	
H5A	0.5788	0.6087	0.3832	0.025*	
H5B	0.5718	0.5046	0.4445	0.025*	
C6	0.44000 (11)	0.51419 (17)	0.38847 (7)	0.0203 (3)	
C7	0.38238 (12)	0.41397 (16)	0.42132 (7)	0.0214 (3)	
C8	0.27640 (12)	0.41627 (18)	0.41733 (8)	0.0268 (3)	
H8	0.2379	0.3530	0.4420	0.032*	
C9	0.22764 (13)	0.51300 (19)	0.37651 (8)	0.0288 (4)	
H9	0.1565	0.5149	0.3742	0.035*	
C10	0.28474 (13)	0.60661 (17)	0.33916 (9)	0.0275 (3)	
C11	0.39017 (12)	0.60955 (16)	0.34741 (9)	0.0268 (3)	
H11	0.4282	0.6766	0.3251	0.032*	

### Atomic displacement parameters (Å^2^)

**Table:**

	*U*^11^	*U*^22^	*U*^33^	*U*^12^	*U*^13^	*U*^23^
Cu1	0.01778 (12)	0.01416 (12)	0.02119 (11)	−0.00129 (6)	−0.00341 (6)	0.00288 (6)
O1	0.0249 (6)	0.0251 (6)	0.0320 (6)	−0.0065 (5)	−0.0100 (5)	0.0112 (4)
O2	0.0206 (5)	0.0312 (6)	0.0306 (6)	−0.0049 (5)	−0.0093 (4)	0.0097 (5)
O3	0.0218 (5)	0.0199 (5)	0.0226 (5)	−0.0030 (4)	−0.0037 (4)	0.0019 (4)
O4	0.0303 (6)	0.0343 (7)	0.0254 (6)	−0.0017 (5)	−0.0107 (4)	0.0048 (5)
O5	0.0266 (6)	0.0260 (6)	0.0251 (6)	0.0021 (5)	0.0062 (4)	0.0086 (5)
O6	0.0264 (7)	0.0411 (8)	0.0532 (8)	0.0104 (6)	−0.0013 (6)	0.0175 (6)
O7	0.0251 (5)	0.0158 (5)	0.0317 (6)	−0.0001 (5)	−0.0054 (5)	0.0033 (5)
N1	0.0135 (5)	0.0169 (6)	0.0187 (5)	0.0001 (4)	−0.0009 (4)	0.0026 (4)
C1	0.0194 (6)	0.0198 (7)	0.0223 (6)	0.0012 (6)	−0.0013 (5)	0.0025 (5)
C2	0.0150 (6)	0.0240 (7)	0.0285 (7)	−0.0019 (6)	−0.0034 (5)	0.0084 (6)
C3	0.0190 (7)	0.0212 (7)	0.0200 (7)	0.0024 (5)	0.0003 (5)	−0.0017 (5)
C4	0.0200 (7)	0.0239 (8)	0.0188 (7)	−0.0013 (6)	−0.0007 (5)	0.0054 (5)
C5	0.0212 (7)	0.0156 (7)	0.0266 (7)	−0.0005 (5)	−0.0002 (6)	−0.0034 (6)
C6	0.0199 (7)	0.0172 (7)	0.0236 (7)	0.0014 (5)	0.0027 (5)	−0.0022 (5)
C7	0.0248 (7)	0.0207 (7)	0.0186 (6)	0.0031 (6)	0.0041 (5)	−0.0009 (5)
C8	0.0231 (7)	0.0309 (9)	0.0263 (8)	−0.0033 (7)	0.0063 (6)	0.0022 (6)
C9	0.0190 (7)	0.0348 (9)	0.0327 (8)	0.0027 (7)	0.0036 (6)	−0.0010 (6)
C10	0.0250 (8)	0.0245 (8)	0.0329 (8)	0.0074 (6)	0.0014 (6)	0.0016 (6)
C11	0.0239 (8)	0.0201 (8)	0.0363 (8)	0.0020 (6)	0.0049 (6)	0.0059 (6)

### Geometric parameters (Å, °)

**Table:**

Cu1—O1	1.9376 (11)	C1—C2	1.522 (2)
Cu1—O3	1.9526 (11)	C2—H2A	0.9700
Cu1—O7	1.9541 (12)	C2—H2B	0.9700
Cu1—N1	1.9929 (12)	C3—C4	1.519 (2)
Cu1—O5	2.3746 (12)	C4—H4A	0.9700
O1—C1	1.2752 (19)	C4—H4B	0.9700
O2—C1	1.2390 (18)	C5—C6	1.503 (2)
O3—C3	1.2903 (18)	C5—H5A	0.9700
O4—C3	1.2302 (18)	C5—H5B	0.9700
O5—C7	1.3818 (19)	C6—C11	1.388 (2)
O5—H5	0.76 (3)	C6—C7	1.390 (2)
O6—C10	1.376 (2)	C7—C8	1.385 (2)
O6—H4	0.86 (3)	C8—C9	1.388 (2)
O7—H7A	0.80 (2)	C8—H8	0.9300
O7—H7B	0.87 (3)	C9—C10	1.385 (2)
N1—C2	1.4810 (18)	C9—H9	0.9300
N1—C4	1.4834 (17)	C10—C11	1.385 (2)
N1—C5	1.4976 (19)	C11—H11	0.9300
			
O1—Cu1—O3	164.41 (5)	O4—C3—O3	123.49 (14)
O1—Cu1—O7	94.69 (5)	O4—C3—C4	119.88 (14)
O3—Cu1—O7	93.01 (5)	O3—C3—C4	116.50 (12)
O1—Cu1—N1	84.90 (5)	N1—C4—C3	110.22 (11)
O3—Cu1—N1	85.11 (5)	N1—C4—H4A	109.6
O7—Cu1—N1	169.72 (5)	C3—C4—H4A	109.6
O1—Cu1—O5	102.29 (5)	N1—C4—H4B	109.6
O3—Cu1—O5	90.00 (5)	C3—C4—H4B	109.6
O7—Cu1—O5	98.05 (5)	H4A—C4—H4B	108.1
N1—Cu1—O5	92.06 (5)	N1—C5—C6	113.23 (12)
C1—O1—Cu1	114.51 (9)	N1—C5—H5A	108.9
C3—O3—Cu1	113.96 (9)	C6—C5—H5A	108.9
C7—O5—Cu1	109.22 (9)	N1—C5—H5B	108.9
C7—O5—H5	112 (2)	C6—C5—H5B	108.9
Cu1—O5—H5	124 (2)	H5A—C5—H5B	107.7
C10—O6—H4	106.8 (16)	C11—C6—C7	118.94 (14)
Cu1—O7—H7A	116.0 (17)	C11—C6—C5	120.25 (14)
Cu1—O7—H7B	109.1 (17)	C7—C6—C5	120.81 (14)
H7A—O7—H7B	109 (2)	O5—C7—C8	123.13 (14)
C2—N1—C4	113.83 (11)	O5—C7—C6	116.67 (13)
C2—N1—C5	109.11 (11)	C8—C7—C6	120.18 (14)
C4—N1—C5	111.37 (11)	C7—C8—C9	120.03 (15)
C2—N1—Cu1	105.92 (9)	C7—C8—H8	120.0
C4—N1—Cu1	106.12 (9)	C9—C8—H8	120.0
C5—N1—Cu1	110.28 (9)	C10—C9—C8	120.18 (16)
O2—C1—O1	124.70 (14)	C10—C9—H9	119.9
O2—C1—C2	118.61 (13)	C8—C9—H9	119.9
O1—C1—C2	116.62 (13)	O6—C10—C11	117.07 (15)
N1—C2—C1	110.04 (12)	O6—C10—C9	123.74 (15)
N1—C2—H2A	109.7	C11—C10—C9	119.17 (15)
C1—C2—H2A	109.7	C10—C11—C6	121.15 (15)
N1—C2—H2B	109.7	C10—C11—H11	119.4
C1—C2—H2B	109.7	C6—C11—H11	119.4
H2A—C2—H2B	108.2		
			
O3—Cu1—O1—C1	36.6 (3)	O2—C1—C2—N1	−161.79 (13)
O7—Cu1—O1—C1	155.93 (11)	O1—C1—C2—N1	21.11 (19)
N1—Cu1—O1—C1	−13.76 (11)	Cu1—O3—C3—O4	179.80 (12)
O5—Cu1—O1—C1	−104.76 (11)	Cu1—O3—C3—C4	3.87 (16)
O1—Cu1—O3—C3	−38.9 (2)	C2—N1—C4—C3	145.89 (13)
O7—Cu1—O3—C3	−158.44 (11)	C5—N1—C4—C3	−90.25 (14)
N1—Cu1—O3—C3	11.43 (10)	Cu1—N1—C4—C3	29.79 (13)
O5—Cu1—O3—C3	103.49 (11)	O4—C3—C4—N1	160.38 (13)
O1—Cu1—O5—C7	130.89 (10)	O3—C3—C4—N1	−23.53 (18)
O3—Cu1—O5—C7	−39.44 (10)	C2—N1—C5—C6	−177.33 (12)
O7—Cu1—O5—C7	−132.48 (10)	C4—N1—C5—C6	56.17 (16)
N1—Cu1—O5—C7	45.67 (10)	Cu1—N1—C5—C6	−61.38 (14)
O1—Cu1—N1—C2	23.76 (9)	N1—C5—C6—C11	−102.16 (17)
O3—Cu1—N1—C2	−144.26 (9)	N1—C5—C6—C7	77.03 (17)
O7—Cu1—N1—C2	−64.4 (3)	Cu1—O5—C7—C8	129.74 (13)
O5—Cu1—N1—C2	125.92 (9)	Cu1—O5—C7—C6	−48.69 (15)
O1—Cu1—N1—C4	145.08 (9)	C11—C6—C7—O5	173.46 (14)
O3—Cu1—N1—C4	−22.93 (9)	C5—C6—C7—O5	−5.7 (2)
O7—Cu1—N1—C4	56.9 (3)	C11—C6—C7—C8	−5.0 (2)
O5—Cu1—N1—C4	−112.75 (9)	C5—C6—C7—C8	175.78 (14)
O1—Cu1—N1—C5	−94.17 (9)	O5—C7—C8—C9	−173.82 (15)
O3—Cu1—N1—C5	97.81 (9)	C6—C7—C8—C9	4.6 (2)
O7—Cu1—N1—C5	177.7 (2)	C7—C8—C9—C10	0.6 (3)
O5—Cu1—N1—C5	7.99 (9)	C8—C9—C10—O6	173.45 (16)
Cu1—O1—C1—O2	−177.60 (12)	C8—C9—C10—C11	−5.2 (3)
Cu1—O1—C1—C2	−0.69 (18)	O6—C10—C11—C6	−174.02 (15)
C4—N1—C2—C1	−145.39 (13)	C9—C10—C11—C6	4.7 (3)
C5—N1—C2—C1	89.54 (15)	C7—C6—C11—C10	0.4 (2)
Cu1—N1—C2—C1	−29.17 (14)	C5—C6—C11—C10	179.58 (15)

### Hydrogen-bond geometry (Å, °)

**Table:**

*D*—H···*A*	*D*—H	H···*A*	*D*···*A*	*D*—H···*A*
O5—H5···O2^i^	0.76 (3)	1.93 (3)	2.6859 (16)	172 (3)
O6—H4···O3^ii^	0.86 (3)	2.00 (3)	2.8442 (18)	168 (2)
O7—H7A···O2^iii^	0.80 (2)	1.97 (2)	2.7261 (16)	157 (2)
O7—H7B···O4^iv^	0.87 (3)	1.83 (3)	2.6794 (18)	163 (2)

Symmetry codes: (i) *x*−1/2, −*y*+1/2, −*z*+1; (ii) −*x*+1/2, *y*+1/2, *z*; (iii) −*x*+3/2, *y*−1/2, *z*; (iv) −*x*+1, *y*−1/2, −*z*+1/2.
